# Barriers and facilitators to writing quality items for medical school assessments – a scoping review

**DOI:** 10.1186/s12909-019-1544-8

**Published:** 2019-05-02

**Authors:** Sowmiya Karthikeyan, Elizabeth O’Connor, Wendy Hu

**Affiliations:** School of Medicine, Western Sydney University, Ainsworth Bldg, Goldsmith Ave, Campbelltown, NSW 2560 Australia

**Keywords:** Assessment, Item writing, Written examination, Quality assurance, Faculty development

## Abstract

**Background:**

Producing a sufficient quantity of quality items for use in medical school examinations is a continuing challenge in medical education. We conducted this scoping review to identify barriers and facilitators to writing good quality items and note gaps in the literature that are yet to be addressed.

**Methods:**

We conducted searches of three databases (ERIC, Medline and Scopus) as well as Google Scholar for empirical studies on the barriers and facilitators for writing good quality items for medical school examinations.

**Results:**

The initial search yielded 1997 articles. After applying pre-determined criteria, 13 articles were selected for the scoping review. Included studies could be broadly categorised into studies that attempted to directly investigate the barriers and facilitators and studies that provided implicit evidence. Key findings were that faculty development and quality assurance were facilitators of good quality item writing while barriers at both an individual and institutional level include motivation, time constraints and scheduling.

**Conclusions:**

Although studies identified factors that may improve or negatively impact on the quality of items written by faculty and clinicians, there was limited research investigating the barriers and facilitators for individual item writers. Investigating these challenges could lead to more targeted and effective interventions to improve both the quality and quantity of assessment items.

## Background

The notion that ‘assessment drives and enhances learning’ [1] emphasises the importance of examinations in ensuring that students graduating from medical school are equipped with the knowledge and skills required to be competent and safe medical practitioners. The questions that constitute written assessments are referred to as ‘items’ and their method of development termed ‘item writing’ [2]. Generating a sufficient number of quality items for assessment on a regular basis is a widespread challenge amongst medical schools [3, 4]. The detrimental effects of poor item quality have been well recognised [5, 6]. Item writing flaws lead to construct-irrelevant variance, affecting pass-fail outcomes for students and, simultaneously, fail to examine what assessors purport to test [5, 7].

A ‘good quality question’ has no simple definition, but for the purposes of this review will be classified as a reliable and valid examination item that obeys accepted item writing guidelines [8–12]; Case and Swanson’s *Constructing written test questions for the basic and clinical sciences* [13], used to create items for the National Board of Medical Examiners (NBME) is perhaps the best recognised in medical education.

Medical school examination questions have conventionally been written by faculty teaching the course. However, meeting the regular demand for new items which have not previously been run with student cohorts has led to strategies such as item modelling, collaborative item banks, computer generated questions and even student written items [3, 14–17], measures which may be beyond the reach of many medical schools. Although significant efforts have been expended in supporting faculty to write better quality and a higher quantity of questions, the evidence which shows that these measures address the root of the problem – the continual need for content experts to contribute to the development of new, quality items – is unknown.

Existing item writing literature consists predominantly of publications focussing on guidelines for writing good quality questions, psychometric analysis of items, comparisons between question formats and studies concerned with the prevalence of item writing flaws as well as their impact on student performance [5–9, 18–24]. There is little research into precisely what makes it so difficult for medical item writers to construct high quality assessment items. The effect of item-writing training is well documented in general education spheres and there is a large body of research into the effectiveness of faculty development programs on improving teaching [6, 25–30]. However, the evidence showing the effectiveness of faculty training at improving medical item writing quality is unknown. The item writing process has been investigated in educational research fields with reference to the challenges as well as the effect of specific quality assurance procedures [25, 27, 28], but these studies did not focus on medical education, which arguably has different challenges.

In medical schools, item writers may include clinicians who are employed primarily to provide patient care, and secondarily to teach. The research on what could motivate clinical content experts to contribute to item writing, when it is not formally part of their position, is not known. The juggle between clinical practice, teaching, research and administration for both clinical and non-clinical academic item writers may influence their ability to provide assessment items. However, without direct evidence, it cannot be assumed that these are the actual facilitators and barriers for item writers to produce good quality questions. We therefore sought to review the evidence to answer the question: what are the barriers and facilitators for current and potential item writers in medical schools to write good quality questions?

In addressing this question, we aimed to identify those factors which could inform the development of evidence based strategies to improve the quality and quantity of items produced in medical schools. We sought to answer this question by systematically reviewing existing literature and adopted the premise that item writing in medical education is a complex and nuanced process that faculty members find challenging for different reasons.

## Methods

The methodology for this scoping review followed Arksey and O’Malley’s framework [31]. In reporting our methods, we were guided by the 2009 PRISMA statement [32].

### Data sources and search strategy

Our search protocol was designed to be broad and inclusive, as there were no universally accepted keywords to cover our research question. We conducted electronic searches of three databases containing peer-reviewed articles (Education Resources Information Centre ERIC, Medline and Scopus). A Google Scholar search was also performed to locate any additional relevant articles. All publications available up until December 2017 were included in the search. The main search terms “item writing” or “item writers” and “medical faculty” were used along with synonyms for “question writing”, medicine, motivation, “faculty training”, barriers, challenges and difficulties. The references of relevant articles found from the electronic searches were then hand searched to find other pertinent literature. Relevant literature on the area known to the researchers and an author search were additional methods of retrieving articles. The initial database searches yielded 1997 articles (excluding duplicates).

### Screening and selection of studies

After duplicates were removed, title screening was performed by SK on all search results and exclusions were made on the basis of irrelevance to the research question. Abstract review and full paper review were then conducted to assess the relevance of articles against inclusion criteria consisting of:

-  ▪ Published in English
-  ▪ Peer-reviewed
-  ▪ Primary research with empirical findings
-  ▪ Centred on medical education in medical schools or undergraduate medical education
-  ▪ Centred on written assessment

**Fig. 1:**
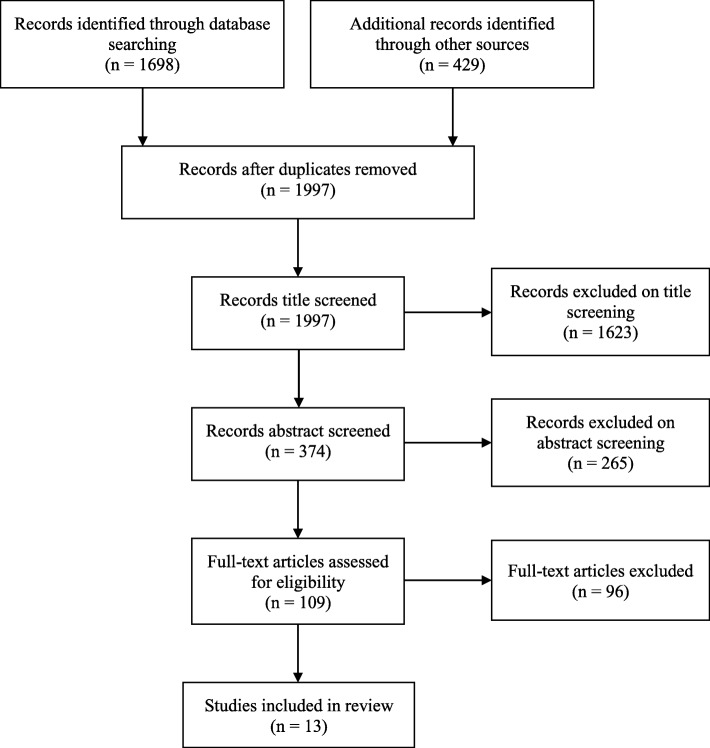
Flowchart of search results

Research in allied health or nursing or general education were excluded. The search and appraisal process is detailed in Fig. 1**.** Title and abstract review yielded 109 articles for full text review, conducted by SK. Two researchers (EO and WH) independently reviewed all articles selected following title and abstract review, as well as a sample of five full text articles to confirm the final sample for full review. The final selection of 13 articles for inclusion were reviewed and agreed upon by all three authors.

Data within each field were then thematically analysed by SK to identify recurrent ideas and concepts as preliminary themes, with the final themes and subthemes developed and agreed by all three researchers through iterative discussion and independent review of selected articles.

## Results

### Study selection

The search strategy yielded 1997 articles in total, of which 13 met the selection criteria and are included in this review.

### Study characteristics

The 13 articles included for this review were published between 1992 and 2017. Of these thirteen, eight are interventional studies while the remaining five are observational in study design (see Tables 1 and 2).

**Table 1:** Data Extraction: Interventional Studies which reported an outcome

Authors	Intervention Type	Setting	Population	Study Design	Outcome	Reference
Abdulghani HM, Ahmad F, Irshad M, Khalil MS, Al-Shaikh GK, Syed S, Aldrees AA, Alrowais N, Haque S	Two day workshop	Saudi Arabia, 2014	Single group, medical faculty	Pre-test, Post-test (Quasi experimental)	Statistically significant improvements in difficulty levels and Bloom’s cognitive levels of items post-intervention.	Abdulghani HM, Ahmad F, Irshad M, Khalil MS, Al-Shaikh GK, Syed S, et al. Faculty development programs improve the quality of Multiple Choice Questions items’ writing. Sci Rep. 2015;5:9556.
Malau-Aduli BS, Zimitat C	Peer review	Australia, 2012	Single group, medical faculty	Pre-test, Post-test	Statistically significant increases in functional distractors, item difficulty, discrimination index and point-biserial correlation.	Malau-Aduli BS, Zimitat C. Peer review improves the quality of MCQ examinations. Assess Eval High Educ. 2012;37(8):919–31.
Naeem N, van der Vleuten C, Alfaris EA	Week long workshop	Pakistan, 2009	Single group, medical and nursing faculty	Pre-test, Mid-test, Post-test (Quasi experimental)	Statistically significant increase in mean scores of items according to a quality checklist.	Naeem N, van der Vleuten C, Alfaris EA. Faculty development on item writing substantially improves item quality. Adv Health Sci Educ Theory Pract. 2012;17(3):369–76.
Shea JA, Poniatowski PA, Day SC, Langdon LO, LaDuca A, Norcini JJ	Item modelling	USA, 1992	Two novice item writers, single group	Post-test	Item modelling is an efficient method for producing good quality test items and is appealing to potential item writers.	Shea JA, Poniatowski PA, Day SC, Langdon LO, LaDuca A, Norcini JJ. An adaptation of item modeling for developing test-item banks. Teach Learn Med. 1992;4(1):19–24.
Wallach PM, Crespo LM, Holtzman KZ, Galbraith RM, Swanson DB	Committee review	USA, 2002	Single group, medical faculty	Pre-test, Post-test	Statistically significant continuous improvement in Quality Assessment Scores (QAS) with the implementation of predetermined guidelines and a committee review process.	Wallach PM, Crespo LM, Holtzman KZ, Galbraith RM, Swanson DB. Use of a committee review process to improve the quality of course examinations. Adv Health Sci Educ Theory Pract. 2006;11(1):61–8.
Iramaneerat C	Three short workshops	Thailand, 2012	Single group, clinical faculty	Pre-test, post-test	Statistically significant increase in post-test item discrimination. Workshops had high satisfaction ratings amongst clinical teachers.	Iramaneerat C. The impact of item writer training on item statistics of multiple-choice items for medical student examination. Siriraj Med J. 2012;64(6):178–182.
Abdulghani HM, Irshad M, Haque S, Ahmad T, Sattar K, Khalil MS	Longitudinal item writing workshops	Saudi Arabia, 2015	Single group, medical faculty	Longitudinal cohort study	Improvement in item quality as determined through item analysis in each successive academic year after the implementation of item writing workshops.	Abdulghani HM, Irshad M, Haque S, Ahmad T, Sattar K, Khalil MS. Effectiveness of longitudinal faculty development programs on MCQs items writing skills: A follow-up study. PLoS One. 2017;12(10):e0185895.
Abozaid H, Park YS, Tekian A	Peer review committee	Saudi Arabia, 2013	Single group, medical faculty	Retrospective cohort study	Statistically significant improvement in item discrimination in medicine, paediatric and surgery examinations. Significant improvement in item difficulty only for the medicine examination.	Abozaid H, Park YS, Tekian A. Peer review improves psychometric characteristics of multiple choice questions. Med Teach. 2017;39 Suppl 1:50–4.

**Table 2:** Data Extraction: Observational Studies

Authors	Setting	Population	Study Design	Summary	Reference
Case SM, Holtzman K, Ripkey DR	USA, 1997	Medical faculty	Case study (uncontrolled trial)	Describes three item models and the item quality and cost outcome for each model.	Case SM, Holtzman K, Ripkey DR. Developing an item pool for CBT: a practical comparison of three models of item writing. Acad Med. 2001;76 Suppl 10:111–3.
Downing SM	USA, 2002	Year 1 basic science exam, single group	Cross-sectional	Suggests that item quality can be poor even with the use of faculty development and item writing guidelines.	Downing SM. Construct-irrelevant variance and flawed test questions: do multiple-choice item-writing principles make any difference? Acad Med. 2002;77 Suppl 10:103–4.
Holsgrove G, Elzubeir M	UK, 1996	Active MBBS examiners	Cross-sectional	Imprecise terms in MCQ items are routine and their interpretation by examiners is highly variable.	Holsgrove G, Elzubeir M. Imprecise terms in UK medical multiple-choice questions: what examiners think they mean. Med Educ. 1998;32(4):343–50.
Jozefowicz RF, Koeppen BM, Case S, Galbraith R, Swanson D, Glew RH	USA, 1998	Three medical schools	Cross-sectional	The overall quality of in-house examination items was low. Mean quality assessment scores were higher for items written by NBME-trained writers than for writers without formal training.	Jozefowicz RF, Koeppen BM, Case S, Galbraith R, Swanson D, Glew RH. The quality of in-house medical school examinations. Acad Med. 2002;77(2):156–61.
Pinjani S, Umer M, Sadaf S	Pakistan, 2008	Aga Khan University medical faculty	Case study	Immediate need for new questions due to hacking of the question database led to measures to rapidly generate new items.	Pinjani S, Umer M, Sadaf S. Faculty engagement in developing an internship entry test. Med Educ. 2015;49(5):540–1.

Findings from these studies were thematically analysed and categorised into facilitators and barriers to item writing. Four themes and eight subthemes emerged and these are discursively presented below (see Table 3**)**.

**Table 3:** Studies categorised by theme and subtheme

Theme	Subtheme	Facilitators and barriers identified	Articles
Faculty Development	Item Writing Training	Facilitator: item writer training Barrier: item writers’ lack of skill Barrier: lack of participation in faculty development for item writing	▪ Abdulghani et al. (2015) [33] ▪ Abdulghani et al. (2017) [34] ▪ Abozaid et al. (2017) [35] ▪ Jozefowicz et al. (2002) [36] ▪ Iramaneerat et al. (2012) [37] ▪ Naeem et al. (2012) [2] ▪ Downing et al. (2002) [7] ▪ Case et al. (2001) [3]
Quality Assurance Procedures	Committee Review and Assessment Blueprinting	Facilitator: peer review Facilitator: assessment blueprinting	▪ Wallach et al. (2006) [38] ▪ Malau-Aduli et al. (2012) [39] ▪ Abozaid et al. (2017) [35] ▪ Jozefowicz et al. (2002) [36] ▪ Pinjani et al. (2015) [4]
	Item Writing Guidelines	Facilitator: utilisation of item writing guidelines Facilitator: item modelling Barrier: lack of utilisation of item writing guidelines	▪ Holsgrove et al. (1998) [40] ▪ Downing (2002) [7] ▪ Abdulghani et al. (2015) [33] ▪ Shea et al. (1992) [41] ▪ Jozefowicz et al. (2002) [36] ▪ Wallach et al. (2006) [38]
Institutional Barriers	Motivation	Facilitator: institutional need for new items	▪ Pinjani et al. (2015) [4] ▪ Case et al. (2001) [3]
	Time constraints and Scheduling	Barrier: poor organisational assessment structure	▪ Wallach et al. (2006) [38] ▪ Jozefowicz et al. (2002) [36]
	Cost and Logistics	Barrier: logistical challenges of organising meetings for item writing Facilitator: cost effectiveness	▪ Case et al. (2001) [3]
Individual Barriers	Motivation	Barrier: lack of motivation	▪ Naeem et al. (2012) [2]
	Time constraints and allocation	Barrier: lack of time allocation for item writing	▪ Shea et al. (1992) [41] ▪ Wallach et al. (2006) [38] ▪ Case et al. (2001) [3] ▪ Jozefowicz et al. (2002) [36]

## Facilitators

### Faculty development

#### Item writer training

Three studies conducted at King Saud University (KSU) found that item quality improved with faculty development [2, 33, 34]. In a 2012 study by Naeem et al.*,* statistically significant increases in mean item quality scores were observed for items produced after training [2]. Abdulghani et al. implemented a workshop for 25 new faculty members, showing an improvement in difficulty index values, discriminating indices and cognitive level of Bloom’s taxonomy post-intervention [33]. A follow-up study, also by Abdulghani et al., studied the effects of faculty development programs on MCQ item quality during successive years in the period between 2012 and 2015 [34]. Statistically significant improvements in discrimination index values and a decrease in item flaws were observed, with each successive year showing greater improvements [34]. However, the study did not acknowledge possible faculty turnover and how this may have affected the training of faculty. The year on year improvement implies cultural change had occurred from regular training, thus raising item quality over time. The longitudinal design of this study provides stronger evidence for faculty training as a facilitator of good quality item writing than before-and-after single intervention studies.

An analysis of 555 examination items from three medical schools in 1998 by Jozefowicz et al. showed a statistically significant increase in the quality of items produced by NBME-trained writers versus writers without training [36]. The authors noted that as item quality was assessed by NBME-trained writers they would be more likely to rate items written by examiners with the same training more highly. However, by our definition that a good quality item is one that follows existing guidelines, the study design is not flawed in this aspect. The NBME training program is internationally recognised in comparison with in-house faculty development implemented in the KSU studies.

Iramaneerat et al. conducted a series of three workshops and evaluated participants’ views on the training. They additionally compared item difficulty and item discrimination between items produced by participants and non-participants [37]. There was a high satisfaction rate amongst participants for appropriateness of content, teaching effectiveness and accomplishment of objectives [37]. However, the results of item difficulty and item discrimination analysis only showed non-significant improvements in these measures after the workshops. Although the evidence for improved item quality was limited, the workshops were perceived by attendees to be beneficial. While participant perception is weak evidence for training effectiveness, it could be argued that increased confidence could result in a greater engagement with item writing, and potentially, motivate them to write more items.

The studies focused on faculty development demonstrate that item writing training is widely used as part of sustained efforts to improve item quality, with more rigorous studies using psychometric analysis to measure training effects on item quality. However, none of the studies measured the effect of faculty training on the *quantity* of good quality items produced after the intervention, and whether there was an improvement in meeting the demand for new questions. As Naeem et al. and Iramaneerat et al. both identified, a limitation of their study designs was that participants were voluntarily recruited and thus were already inherently inclined or motivated to engage with measures to improve item writing quality and quantity [2, 37].

### Quality assurance procedures

#### Committee review and assessment blueprinting

Quality assurance procedures such as a peer review committee that screens potential items and offers feedback to writers are often recommended and were reported to improve item quality [35, 38, 39]. For example, Pinjani et al. used pre-established guidelines and assessment blueprints as part of their item writing intervention [4]. In a study by Wallach et al., an item quality analysis of three examinations by NBME staff members who were blinded to the year of origin was conducted. They reported significant increases in the Quality Assessment Score of items from the two papers written after the establishment of guidelines and a committee review process [38]. A similar study at an Australian medical school also found an increase in psychometric quality of items after the implementation of the peer review process in conjunction with assessment blueprinting and other quality assurance processes [39]. A retrospective cohort study by Abozaid et al. of two consecutive years (2012–2013) was used to analyse the effects of an assessment peer review program for different specialties [35]. While there was a significant improvement in item discrimination in medicine, paediatric and surgery examinations, there was significant improvement in item difficulty only for the medicine examination [35]. Perhaps more pertinent to facilitating good quality item writing is the feedback provided by the review committee to item writers to improve their understanding of item writing processes and increase the quality of the items produced [35]. Whether being a member on the committee in and of itself facilitates item writing was not considered in these studies.

#### Item writing guidelines

There is much literature that provides item writers with good quality question construct guidelines that aim to improve item quality [7, 33]. Both Jozefowicz et al. and Wallach et al. recommended the implementation of pre-established guidelines to facilitate quality item writing [36, 38]. However, as Holsgrove and Elzubeir 1998 noted, encouraging the actual use of such guidelines by medical item writers can prove to be challenging [40]. For example, despite the availability of guidelines, one in three items in a basic science test were found to be flawed in Downing’s 2002 study where items underwent psychometric analysis and were rated by blinded assessors for adherence to item-writing principles [7]. Downing suggested that item-writing training would improve use of guidelines and act to decrease item flaws, and also highlighted the importance of training measures with long term follow up and feedback to writers [7].

Inconsistent interpretation of commonly used terms among item writers is yet another barrier to the production of good quality questions. One UK survey of 70 examiners involved in the MCQ writing and approval process for medical schools reported that there were discrepancies in the way participants viewed terms, for example, the word ‘always’ was interpreted by 51 participants to mean 100% of the time while 3 examiners believed it meant 80% of the time [40]. Many item writing guidelines recommend against the use of absolute terms [9, 10, 13, 18], but in practice these guidelines are not always followed. This suggests that a barrier to writing good quality items is lack of understanding and/or use of item writing guidelines, resulting in unintended violation of assessment best practice principles. Shea et al. proposed the use of standard item shells as a solution to the difficulties of styling and formatting questions, allowing writers to concentrate on issues of content instead [41].

## Barriers

### Institutional factors

#### Motivation

Although there is a lack of empirical research into what motivates item writers to be part of the assessment writing process and the role this may or may not play in the quality of the items that they produce, some studies did suggest that it is an area for consideration. One case study describes the rapid production of good quality items on short notice due to a hacking of the university item bank [4]. A retreat for faculty was initiated and 100 new test items were constructed, reviewed and approved. The success of the process implies that institutional motivation, in the form of immediate threat and the need for new test items, can facilitate innovative and efficient processes for item writing and produce good quality items. This notion is supported by a case study of different models for item writing, which also suggests that immediate pressure for new items created an environment in which inventive item writing measures were necessary [3].

#### Time constraints and scheduling

The challenges of allocating time for item writing, item writing training and committee review meetings for academics and clinicians who may have other roles and commitments are obvious [42, 43]. Indeed, Jozefowicz et al. (2002) suggest that a possible cause of poor quality items is that faculty spend little time on the item construction process [36]. Similarly, Wallach et al. (2006) highlights that the amount of preparation that goes into creating teaching materials and lectures far outweighs the time allocated to writing assessments which test the very concepts faculty members make great efforts to teach [38]. Both Jozefowicz et al. (2002) and Wallach et al. (2006) make recommendations for organisational improvements such as preparing examinations and setting committee review dates weeks in advance to decrease the haphazard approval of assessment items [36, 38].

#### Cost and logistics

The logistics and financial cost of implementing an institutional process for producing quality assured items are additional areas of difficulty [3]. The best methods are not always the most economically or logistically feasible and this may compromise the ability of institutions to facilitate good quality item writing. Case et al. compared three models of item writing to evaluate the cost effectiveness and item yield of each process, concluding that the traditional test committees model produced high quality items at a reasonable financial cost [3].

### Individual factors

#### Motivation

The Naeem et al. study on the effect of faculty training raised the issue that such programs require motivation on the part of individuals to attend. However, this was not an outcome that was measured [2]. A number of papers implicitly refer to motivation as a likely barrier to writing good quality examination papers, but do not directly assess motivation or what factors might promote engagement in item writing [2, 36–38].

#### Time constraints and allocation

Unsurprisingly, item writers find question writing processes that require less time commitment more appealing [3]. Efforts have been made to develop procedures that have a high yield of good quality items, with time and cost as factors for consideration [3]. One such method is item modelling, which involves deconstructing an existing item stem into its constituent elements and writing new items based on these elements. For example, altering the item stem itself to create another item and/or use of an item shell during item construction [41]. Case et al. found that the traditional standing test committee model had the highest yield of good quality items for a combined staff time (academic/clinical and administrative) per approved item of 1.5 h [3]. As mentioned earlier, authors have noted that allocating time to item writing is often not considered by teachers as part of preparing teaching materials [36].

## Discussion

Our review identified few research papers which directly investigated the barriers and facilitators to quality item writing. There were, however, studies which attempted to measure the outcomes from interventions to improve item writing quality. The selected studies could be categorised into i) studies that attempted to empirically measure the barriers and facilitators as outcomes of the interventions, or ii) studies that provided implicit evidence. An example of the former is the survey of item writers conducted by Holsgrove et al. [40], which identified discrepancies in language construction amongst current item writers as a barrier to good quality item writing. An example of the latter is a psychometric analysis of items by Downing et al. (2002) which suggested that item writer training was lacking. Better studies measured the psychometric qualities of items pre and post intervention to assess improvement in item quality, rather than participant perceptions and confidence [2, 7, 33–35, 37, 39].

The studies in this review have tended to assume that the problem of writing good quality items is due to a lack of skill amongst writers; this is highlighted by seven of the thirteen review articles focussing on faculty development programs [2, 3, 33–37]. We suggest that there are other factors involved and in particular, a potential reason for the poor quality and quantity of items is lack of motivation or structural constraints at both an individual and institutional level. At the level of individuals, Self-Determination Theory has been used to understand what motivates educators to teach or engage in scholarship [43, 44], and its components of autonomy, competence and relatedness may be applicable to item writing. For example, faculty training may improve confidence and perceived competence and thus greater willingness to contribute items, although our review suggests that short term psychometric analyses of the items produced may not capture this desirable outcome. Conversely, Sorinola et al. (2015) delved into the role of motivation and engagement *on*, rather than resulting from, the effectiveness of faculty development programs [45]. However, this study did not examine item writing training. There is a lack of theoretically informed research designed to understand the nuances and attitudes towards item writing held by those who are called upon to write items.

While item writing skills, motivation and the benefits of peer review appear to be important at the individual level, there are additional barriers at an institutional level. Several papers imply that a lack of time allocation for item writing and associated training and meetings is a barrier, though this was not directly measured [3, 36, 38, 41]. Other possible barriers include the level of importance placed on assessment at medical schools and whether there is an organisational structure and governance with leading academics on assessment who can guide item writers with clear timeline and expectations for item generation [36, 38]. For some content experts, item writing is not an explicit role in their job description nor is it regularly evaluated in teaching performance evaluations and thus the lack of formal recognition is another possible barrier.

This review has identified motivation, lack of time, variations in use and understanding of terms and institutional difficulties with costs and logistics as barriers to writing good quality questions. Some studies imply that implementation of faculty training, quality assurance procedures including assessment blueprinting, peer review of test items and use of item writing guidelines facilitate the construction of reliable and valid assessment items [2–4, 7, 33–41]. However, their effectiveness in medical schools needs to be further explored and the ability and motivation of clinical teachers and educators to access such training is frequently limited. Although guidelines for item writing are numerous and have been argued to be useful tools to produce good quality questions, common item writing flaws persist in many high stakes examinations despite access to such guides [7, 36, 40]. Ensuring the use of existing guidelines for item writing is similarly challenging. Although item writers may understand the need for their use, making their use a reality appears to pose ongoing challenges.

Implementation of faculty development programs targeting item writing is one institutional intervention that has been found to improve item quality [2, 3, 33–37]. However, an improvement in item quality may still not produce enough high quality items at the rate required for medical programs. There is also an absence of research on how best to engage potential item writers in faculty development activities and this is an area for further investigation.

Our search has not revealed research that directly addresses the core issue of exactly *why* there is a difficulty in writing good quality items. The studies in this review did not confirm that sufficient new, high quality items were produced as a result of the interventions described. In the absence of evidence identifying the underlying difficulties, the design of any interventions may not be as effective as they should. Student authored items, collaborative item banks and automated item generation have been investigated as strategies to increase item production [15–17]. However these strategies essentially circumvent, rather than reduce the barriers to item writing from content experts and teachers. While there is some evidence for item modelling, the results are limited in the diversity of new items produced. We were not able to draw firm conclusions about the effect of quality assurance procedures and item modelling on addressing individual and institutional barriers to item writing.

### Study limitations

We conducted a systematic search through online databases to retrieve articles that were relevant to the research question and used explicit criteria to select the studies for review. There was a lack of published literature directly addressing the review question, with only 13 of the 1997 articles retrieved meeting the pre-determined selection criteria. Most of these papers also did not directly address our review question, and the findings were interpreted to identify the underlying assumptions and measures of barriers and facilitators. Alternative interpretations of the studies may have resulted with a different research team. However, we used pre-determined inclusion criteria, were deliberately broad in our search, and used a process of independent review, checking and re-review to ensure that our findings were transparent and reproducible. We did not formally appraise the quality of the study designs, seeking only to identify primary research with empirical measures of barriers and facilitators to item writing. We acknowledge that newer concepts of validity, suggesting that validity is not a static construct (for example, Kane’s validity [46, 47]) have not been adopted by the authors of papers in our review. Addressing the factors identified in our review in the light of these newer understandings is likely to improve the validity of test items in the future. Due to the limited evidence found we make no absolute claims or strong recommendations about appropriate strategies or interventions to improve item quality and quantity in medical schools.

## Conclusions

Faculty development, quality assurance processes, individual barriers and institutional barriers have been identified as barriers and facilitators to quality item writing in medical schools. However, our review of the primary research has highlighted that the specific challenges which individual item writers face is largely unknown. While there is evidence that faculty development can assist, how best to engage potential item writers in such interventions and to promote institutional attention to item quality is not well researched. Future research could explore the complexities of item writing, focussing on the experiences and attitudes of the writers themselves and how institutional practices may encourage or discourage engagement in measures to improve assessment quantity and quality.

## Abbreviations

MCQ: Multiple choice question
NBME: National Board of Medical Examiners
